# Ki-67 gene expression

**DOI:** 10.1038/s41418-021-00823-x

**Published:** 2021-06-28

**Authors:** Sigrid Uxa, Paola Castillo-Binder, Robin Kohler, Konstanze Stangner, Gerd A. Müller, Kurt Engeland

**Affiliations:** 1 Molecular Oncology, Medical School, University of Leipzig, Leipzig, Germany; 2 Present Address: Ludwig-Maximilians-Universität München, Anatomische Anstalt, Munich, Germany; 3 Present Address: Department of Chemistry and Biochemistry, University of California, Santa Cruz, CA USA

**Keywords:** Tumour biomarkers, Tumour-suppressor proteins, Cell biology, Gene expression, Chromatin

## Abstract

Ki-67 serves as a prominent cancer marker. We describe how expression of the *MKI67* gene coding for Ki-67 is controlled during the cell cycle. *MKI67* mRNA and Ki-67 protein are maximally expressed in G_2_ phase and mitosis. Expression is dependent on two CHR elements and one CDE site in the *MKI67* promoter. DREAM transcriptional repressor complexes bind to both CHR sites and downregulate the expression in G_0_/G_1_ cells. Upregulation of *MKI67* transcription coincides with binding of B-MYB-MuvB and FOXM1-MuvB complexes from S phase into G_2_/M. Importantly, binding of B-MYB to the two CHR elements correlates with loss of CHR-dependent *MKI67* promoter activation in B-MYB-knockdown experiments. In knockout cell models, we find that DREAM/MuvB-dependent transcriptional control cooperates with the RB Retinoblastoma tumor suppressor. Furthermore, the p53 tumor suppressor indirectly downregulates transcription of the *MKI67* gene. This repression by p53 requires p21/CDKN1A. These results are consistent with a model in which DREAM, B-MYB-MuvB, and FOXM1-MuvB together with RB cooperate in cell cycle-dependent transcription and in transcriptional repression following p53 activation. In conclusion, we present mechanisms how *MKI67* gene expression followed by Ki-67 protein synthesis is controlled during the cell cycle and upon induction of DNA damage, as well as upon p53 activation.

## Introduction

The Ki-67 protein is a prominent proliferation marker used in pathology [1, 2]. Ki-67 was first identified as an antigen for a monoclonal antibody detected in the nuclei of proliferating cells [1, 3]. For a long time, the function of this protein remained obscure, even after antibodies directed against Ki-67 were already established tools in cancer diagnostics. The sole relevant feature of Ki-67 was its absence in resting cells and its expression when cells were proliferating [2, 4]. With the establishment of the Ki-67 labeling index, Ki-67 has developed into a standard in diagnosis and prognosis assessment of cancer patients [2]. Complemented by cancer tissue-specific markers, Ki-67 serves as a general indicator for diagnosis and prognosis. The diagnostic procedure for breast cancer with assessment of estrogen receptor, HER2, progesterone receptor, and Ki-67 represents one such example [5].

In contrast to its diagnostic importance, results on the function of Ki-67 were published only recently. The protein is expressed as 320 and 359 kDa isoforms derived from differentially spliced mRNA variants encoded by the human *MKI67* gene [2]. Both Ki-67 isoforms serve in a similar function as a surfactant to keep mitotic chromosomes apart after breakdown of the nuclear envelope. By binding to protein phosphatase 1, Ki-67 contributes to the formation of the perichromosomal protein compartment [6]. Furthermore, Ki-67 facilitates chromosome attachment to the mitotic spindle and individual chromosome mobility through covering the surface of chromosomes and creating a membrane-independent intracellular compartment [7]. Through covering chromosomes and forming the perichromosomal region, Ki-67 also contributes to organizing heterochromatin [8]. These Ki-67 features are also responsible for its role in excluding large cytoplasmic molecules such as ribosomes from nuclei that newly form at the end of mitosis [9]. At least in some cell culture systems, depletion of Ki-67 slows down S phase entry [10]. A recent report shows that Ki-67 supports several steps in carcinogenesis [11].

In contrast to the importance of Ki-67 protein expression for cancer diagnostics, only limited information is available on how the *MKI67* gene is expressed. It has been shown that the Ki-67 protein is degraded in G_1_ phase and upon cell cycle exit by the proteasome [12], and that its mRNA is expressed in a cell cycle-dependent manner [13, 14]. Early reports indicated that overexpression of E2F1 could directly or indirectly increase *MKI67* mRNA levels [15]. Furthermore, the transcription factor Sp1 was implicated in activating *MKI67* transcription [16]. In several meta-analyses of genome-wide chromatin immunoprecipitation (ChIP) data, we have observed that the *MKI67* gene is bound by components of the DREAM transcriptional repressor complex [17–19]. DREAM binds to CHR, CDE/CHR, E2F, and E2F/CLE transcription factor-binding sites [20–23]. DREAM contains proteins related to RB (Retinoblastoma tumor suppressor protein, *RB* or *RB1* gene) and members of the E2F transcription factor family, as it is composed of RBL1 (p107) or RBL2 (p130), E2F4 or E2F5, and DP proteins [24, 25]. In addition to these factors, the DREAM repressor complex contains the MuvB core proteins LIN9, LIN37, LIN52, LIN54, and RBBP4 [24, 26]. Functionally, the repressor can turn into a transcription activator by switching the proteins associating with the MuvB core complex to B-MYB and FOXM1 [19, 22, 24, 27–30]. In addition to these observations on DREAM component binding, we also compiled data from many studies showing that *MKI67* mRNA is downregulated when the transcription factor and tumor suppressor p53 is activated [17–19]. DREAM binding to target promoters and indirect transcriptional repression by p53 are connected through the p53-p21-DREAM pathway [19, 31]. Starting with these initial observations, we analyzed transcriptional regulation of the *MKI67* gene.

Here we report that *MKI67* mRNA and Ki-67 protein expression during the cell cycle depend on DREAM/MuvB complexes that cooperate with RB. The same cooperation also controls *MKI67* and Ki-67 expression in response to p53 activation and upon induction of DNA damage.

## Materials and methods

### Knockout cell lines

HCT116 wild type (WT), HCT116 *p53*^−/−^, and HCT116 *p21*^−/−^ cells were a generous gift from Bert Vogelstein [32]. NIH3T3 *Lin37*^−/−^ and NIH3T3 *Rb*^−/−^, and NIH3T3 DKO (Lin37/Rb double knockout), as well as HCT116 *Lin37*^−/−^ and NIH3T3 *Rb*^−/−^ cell lines were generated using a CRISPR/Cas9 nickase approach [33, 34].

### Cell culture and drug treatment

Human glioblastoma T98G cells, human epithelial-like immortalized retina hTERT-RPE-1 cells, human immortalized foreskin hTERT-BJ cells, mouse NIH3T3 fibroblasts, human bone osteosarcoma U2OS cells, human colorectal carcinoma HCT116 WT, HCT116 *p53*^−/−^, and HCT116 *p21*^−/−^ cells were cultivated in Dulbecco’s modified Eagle’s medium (DMEM; Lonza). DMEM was supplemented with 10% fetal calf serum (FCS; Biochrom) and 1% penicillin/streptomycin (Sigma-Aldrich). Standard growth conditions with 37 °C and 10% CO_2_ were chosen. For serum starvation, FCS was removed from the medium for 72 h. For restimulation, 20% FCS were added to the medium. For p53 activation, the cells were treated with either 10 µM nutlin-3a (Cayman Chemicals) or 0.2 µg/ml doxorubicin (Doxo; Medac GmbH) for 48 h. Flow cytometry analyses of serum-starved NIH3T3 wt, *Lin37*^−/−^, *Rb*^−/−^, and DKO cells have been described earlier [33]. PCR-based tests for mycoplasma contamination were performed as described earlier [35].

### Sequence alignment and ChIP tracts

The UCSC Genome Browser database was employed to retrieve and align genomic sequences [36]. To analyze published ChIP data, we searched the ENCODE database and compared profiles using the UCSC Genome Browser [36, 37]. To compare different ChIP-sequencing tracks and different genome loci, the vertical viewing ranges were consistently set to a minimum of 0 and a maximum of 20.

### Flow cytometry

DNA content of propidium iodide-stained cells was analyzed by flow cytometry (LSR II, Becton Dickinson) as described before [20]. The software FlowJo (Becton Dickinson) was used for cell cycle phase analysis.

### Semi-quantitative real-time PCR and transcription start site mapping

Total RNA was isolated using TRIzol Reagent (ThermoFisher Scientific) according to the manufacturer’s protocol. Reverse transcription and quantitative PCR were combined in a one-step quantitative PCR (qPCR) reaction using the QuantiTect SYBRGreen PCR Kit (Qiagen). Samples were measured on an ABI 7300 Real-Time PCR System (Applied Biosystems). All primer sequences are listed in Suppl. Table 1.

### SDS-PAGE and western blotting

For SDS-polyacrylamide gel electrophoresis (SDS-PAGE) and western blotting, standard in-house protocols were followed as described earlier [38]. For protein detection, the following antibodies were employed: Ki-67 (MIB1, DAKO), β-Actin (A5441, Sigma-Aldrich), LIN37 (T3, custom-made at Pineda Antikörper-Service, Berlin, Germany) [39], LIN54 (A303-799A, Bethyl Laboratories), LIN9 (ab62329, Abcam), p130 (RBL2, D9T7M; Cell Signaling Technologies), KIF23 (MKLP-1, sc-136473; Santa Cruz Biotechnology), p53 (Ab-6, DO-1; Merck/Calbiochem), p21 (Ab-1, EA10; Merck/Calbiochem), E2F1 (C-20, sc-193; Santa Cruz Biotech.), E2F1 (A300-766A, Bethyl Laboratories), E2F4 (C-20, sc-866; Santa Cruz Biotech.), RB (D20, No. 9313; Cell Signaling Technologies), and NF-YA (G-2, sc-17753; Santa Cruz Biotech.). The monoclonal B-MYB LX015.1 antibody (hybridoma media 1 : 5) was a kind gift from Roger Watson.

Protein quantification from western blottings was performed with the LabImage 1D software (Kapelan Bio-Imaging, Leipzig, Germany).

### Plasmids

A 596 bp-long promoter region of the *MKI67* gene was amplified from human genomic DNA extracted from HFF cells and cloned into the pGL4.10 vector backbone. Site-directed mutation of promoter elements was performed by following the QuikChange Site-Directed Mutagenesis protocol (Stratagene). All primers used are listed in Suppl. Table 1. Expression plasmids for Lin37 and Rb were described earlier [33, 40]. The plasmid for expression of a short hairpin RNA (shRNA) targeting mouse B-Myb was a kind gift from Kenneth Boheler (pSuper.neo, Oligoengine; target sequence: 5′-GGTGCGACCTGAGTAAATT-3′) [41]. As a control, a plasmid expressing an shRNA-targeting green fluorescent protein (pSuper.neo; target sequence 5′-GATGTTGTCAGTGAGAGAG-3′) was created.

### Luciferase promoter reporter assays and siRNA transfections

Luciferase reporter assays were performed with extracts of transfected synchronized NIH3T3 or RPE-1 cells as described before [20]. Twenty-four hours post transfection with GeneJuice (EMD Millipore), cells were washed twice with phosphate-buffered saline and DMEM w/o FCS was added. For rescue experiments, Lin37^−/−^, Rb^−/−^, and Lin37^−/−^;Rb^−/−^ (DKO) NIH3T3 cells were plated in 12-well plates (25,000 cells per well) and transfected with 150 ng of promoter reporter plasmids along with 200 ng of constructs expressing WT Lin37 and Rb. To analyze the response of the *MKI67* promoter reporter constructs to knockdown of B-Myb, NIH3T3 cells were plated in 24-well plates at a density of 12,500 cells per well. Each well was transfected with 100 ng reporter construct, 200 ng shRNA-expressing plasmids, 20 ng pGL4.70[hRluc] (Promega), and 1 µl FuGENE (Promega). Cells were collected 48 h post transfection.

To analyze the response of the *MKI67* promoter reporter constructs to B-MYB knockdown, cells were transfected with 100 ng promoter reporter construct, 40 ng pGL4.70[hRluc] (Promega), as well as 0.8 µl DharmaFECT Duo (Dharmacon, Inc.), plated in 24-well plates at a density of 25,000 cells per well. After 24 h, medium was changed and cells were transfected with 10 nM or 25 nM small interfering RNA (siRNA) (ON-TARGETplus Human MYBL2 SMARTPool siRNA and ON-TARGETplus Non-targeting Control Pool; from Dharmacon, Inc.) and 0.75 µl Lipofectamine RNAiMAX (ThermoFisher). Cells were collected 24 h post siRNA transfection.

### DNA affinity purification

DNA affinity purification assays were performed as described earlier [20, 42]. Briefly, biotinylated DNA probes were amplified via PCR from respective promoter reporter pGL4.10 plasmids. All primer sequences and plasmids are listed in Suppl. Table 1. DREAM and MMB complex components were purified from nuclear extracts from serum-starved T98G or NIH3T3 cells, or from proliferating HeLa cells with the biotinylated DNA probes. Bound proteins were separated by SDS-PAGE and detected by western blotting.

### Chromatin immunoprecipitation

ChIP assays were described earlier [23, 43]. The following antibodies were used to precipitate DREAM, MMB, and E2F complex components: E2F4 (C-20, sc-866; Santa Cruz Biotech.) [44], E2F1 (C-20, sc-193; Santa Cruz Biotech.), E2F2 (C-20, sc-633; Santa Cruz Biotech.), E2F3 (C-18, sc-878; Santa Cruz Biotech.), LIN37 (T1, custom-made at Pineda Antikörper-Service, Berlin, Germany), BMYB (N-19, Santa Cruz Biotech.), FOXM1 (C-20, sc-502, Santa Cruz Biotech.) [33], and NF-YA (G-2, sc-17753; Santa Cruz Biotech.). The E2F3 antibody was directed against the C terminus of the protein and did not distinguish between E2F3A and E2F3B, which differ in their N-terminal domain [45]. As a negative control for the ChIP experiments, rabbit IgG was used (Rabbit IgG Isotype control, No. 02-6102; ThermoFisher Scientific).

## Results

### Ki-67 is maximally expressed in G_2_ phase and mitosis

**Fig. 1:**
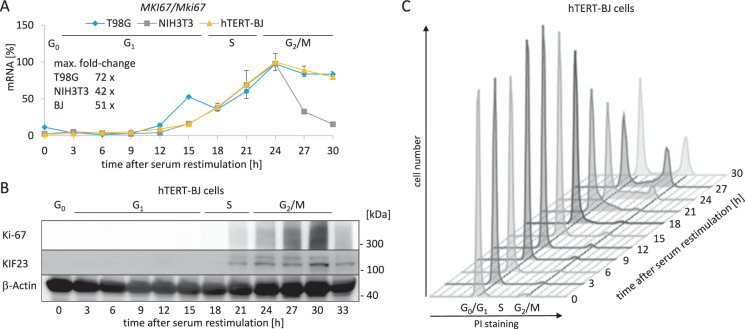
Ki-67 expression on the mRNA and protein levels reaches a maximum in G_2_/M. **A** Expression of *MKI67/Mki67* mRNA over the course of one cell cycle. Human T98G (blue), mouse NIH3T3 (gray), and human hTERT-BJ cells (yellow) were arrested in G_0_ by serum starvation for 72 h. To stimulate the cells to re-enter the cell cycle, FCS was added to the medium. Cells were collected every 3 h after serum restimulation. Each sample was divided into three aliquots for RNA and protein extraction, and for flow cytometry analyses. Relative mRNA levels from *MKI67/Mki67* genes were quantified using real-time RT-qPCR. Mean values ± SD of two technical replicates for each time point are given. The maximum fold-change in *MKI67/Mki67* mRNA levels was calculated for each cell line. Approximations of cell cycle phase distribution for hTERT-BJ cells were deduced from flow cytometry analyses in **C** and are indicated above the graphs. **B** Protein levels of Ki-67 in hTERT-BJ cells used in **A**, **B**, and **C** over the course of one cell cycle were analyzed by western blot analysis. One representative experiment from three biological replicates is shown. KIF23 and β-actin proteins served as cell cycle and loading controls, respectively. Approximate cell cycle phases are indicated. **C** Flow cytometry analyses of propidium iodide (PI) staining of hTERT-BJ cells analyzed in **A** and **B**. Quantification of Ki-67 protein expression of two biological replicates is displayed in Suppl. Fig. 1.

We measured *MKI67* mRNA expression in synchronized human T98G and hTERT-BJ cell lines, as well as in mouse NIH3T3 cells (Fig. 1A). Serum-starved cells were released from the G_0_/G_1_ arrest by serum addition and were collected at several time points during the cell cycle. In all cell lines analyzed, the mRNA levels in G_0_/G_1_ are low and begin to rise in late G_1_ phase and with entry into S phase. The maximum of expression is reached in G_2_/M phases (Fig. 1A).

Next, we measured Ki-67 protein levels in resting hTERT-BJ cells and from cell populations enriched in the different phases of the cell cycle (Fig. 1B, C and Suppl. Fig. 1). The expression of Ki-67 protein was not detectable for the first 18 h following restimulation of cells to enter the cell cycle. The protein was initially detected after 21 h of restimulation, corresponding to S phase. Ki-67 protein levels reached maximal expression at about 30 h, with most cells in G_2_ phase or mitosis. Ki-67 protein followed *MKI67* mRNA expression, lagging by about 5 h. Expression of the KIF23 protein served as a positive control for cell cycle-dependent expression (Fig. 1B). *KIF23* is an established DREAM-CHR target gene with an expression maximum in G_2_/M [46].

### Identification of phylogenetic conserved putative regulatory sites and the transcription start in the *MKI67* gene

**Fig. 2:**
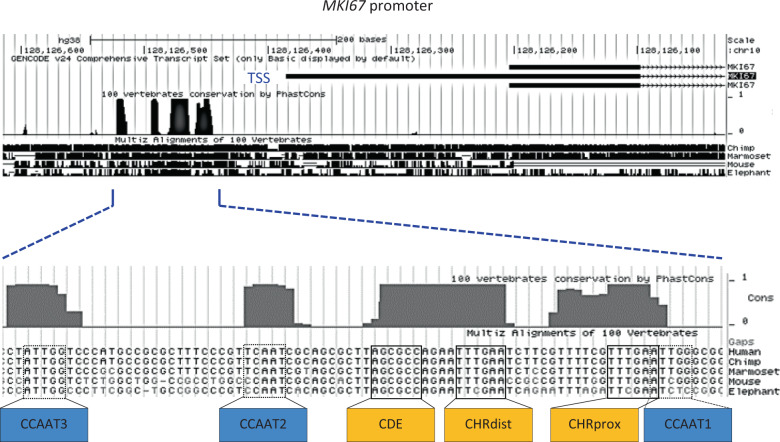
Identification of phylogenetic conserved putative regulatory sites and the transcription start in the *MKI67* gene. A DNA segment spanning ~0.6 kb upstream from the first *MKI67* intron of the human gene (genome version hg38) and surrounding the transcriptional start site (TSS) was aligned to 100 vertebrate genes using the UCSC Genome Browser. The most conserved region of about 100 nucleotides is displayed in detail giving a sequence alignment of the human, chimpanzee, marmoset, mouse, and elephant gene regions. Transcriptional elements are highlighted and named relative to their position to the *MKI67* coding region: CCAAT-boxes (CCAAT1, 2, and 3), proximal cell cycle genes homology region CHR (CHRprox), cell cycle-dependent element (CDE), and distal CHR (CHRdist).

We were interested in elucidating how transcription of the *MKI67* gene is regulated. As phylogenetic conservation can locate important regulatory DNA segments, we searched the *MKI67* promoter for conserved potential transcription factor-binding sites. Analysis of the region upstream of *MKI67* exons with the UCSC Genome Browser yielded several conserved elements (Fig. 2). We mapped the transcription start site (TSS) to the 5′-end of the UTR-1 transcript, which begins just downstream of the conserved region (Suppl. Fig. 2). Also, there is no conserved TATA-box in the *MKI67* upstream region (Fig. 2), which is often observed in promoters of cell cycle genes [21].

Interestingly, two CHR elements are conserved in the *MKI67* upstream region (Fig. 2). This is unprecedented, as functional CHR sites have been discovered so far only as single elements [23]. Both *MKI67* sites are identical with the canonical CHR sequence 5′-TTTGAA-3′ [23]. CHRprox, the site found proximal to the coding region, is localized 12 nucleotides downstream from the distal element (CHRdist). Upstream of CHRdist and separated by a four-nucleotide spacer, a conserved CDE site is located. Overlapping with the CDE, an element similar to an E2F site is conserved (Fig. 2). However, with the sequence 5′-TTAGCGCC-3′, this element does not conform to the E2F-binding consensus 5′-TTTc/gGCGCc/g-3′ E2F [47].

Furthermore, we identified three conserved CCAAT-boxes [48]. CCAAT1 and CCAAT3 are oriented in reverse orientation relative to the TSS (Fig. 2). With the sequence 5′-TCAAT-3′, CCAAT2 deviates from the consensus for binding of NF-Y [48]. CCAAT1 overlaps by one nucleotide with CHRprox (Fig. 2).

### A 596 bp DNA fragment comprising evolutionary conserved transcription factor-binding sites can direct cell cycle-dependent transcription of the *MKI67* gene

**Fig. 3:**
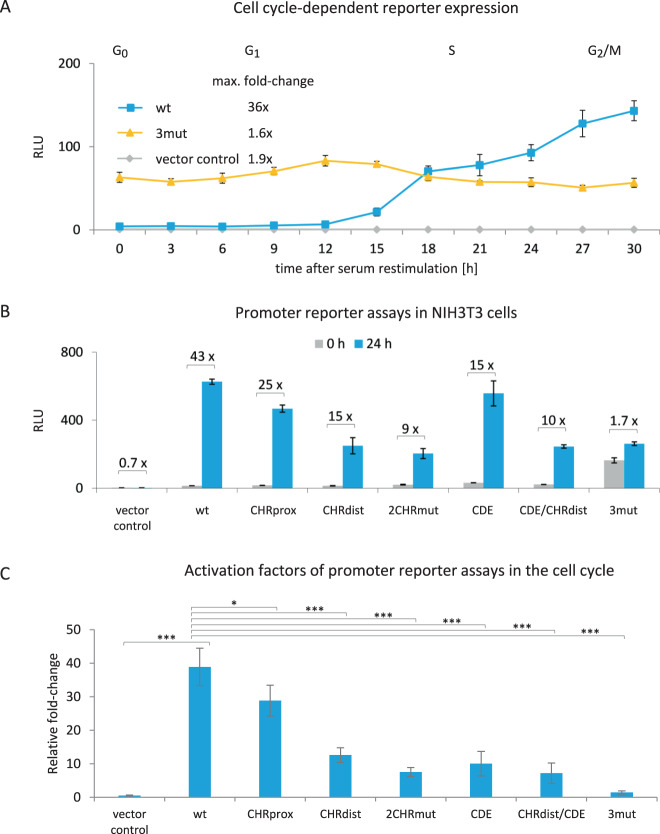
Two CHR sites and one CDE control *MKI67* cell cycle-dependent transcriptional regulation. Luciferase reporter promoter assays from transfected cells that were serum-starved and restimulated by serum addition to the medium. Transfected plasmids were the empty reporter vector pGL4.10 (vector control) and wild-type or mutant reporter plasmid constructs based on a 596 bp *MKI67* fragment including the TSS (see Fig. 2 and Suppl. Fig. 2 for position): wild-type (wt), mutant of the proximal CHR (CHRprox), mutant of the distal CHR (CHRdist), mutant of both CHR elements (2CHRmut), CDE mutant (CDE), a combination of mutations in CHRdist and CDE (CDE/CHRdist), and mutation of the three sites CHRprox, CHRdist, and CDE (3mut). Relative light units (RLUs) were calculated as the ratio of firefly luciferase activity from the promoter reporter constructs to the *Renilla* luciferase activity from a cotransfected control plasmid lacking a promoter. **A** Promoter reporter activity in different phases of the cell cycle. NIH3T3 cells were arrested in G_0_ through serum starvation for 72 h. To stimulate the cells to re-enter the cell cycle, serum was added to the medium. Luciferase assays from NIH3T3 cells transfected with vector control (gray), wt (blue), or 3mut (yellow) *MKI67* firefly reporter promoter constructs. Cells were collected every 3 h after serum restimulation and RLUs were measured from three technical replicates for each time point. One representative experiment from two biological replicates is shown. The maximum fold-change for each construct was calculated. Approximate cell cycle phases were assessed by flow cytometry and are given above the graph. **B** NIH3T3 cells were serum-starved for 72 h (0 h, gray) and restimulated by serum addition (blue) for 24 h. One representative experiment from three biological replicates is shown. Cells were collected and RLUs were measured as technical duplicates. The relative fold-change in RLUs from 0 to 24 h for each promoter construct was calculated and is given above the columns. For all graphs, mean values ± SD are given. **C** Compilation of relative activation factors for each tested plasmid from experiments as shown in **B**. Averages of activation factors of RLUs 24 h after restimulation over RLUs at 0 h before stimulation. Activation factors yield a rate of activation for each promoter construct in the cell cycle. Results are from *n* ≥ 3 biological replicates for each plasmid examined. Significance was tested via an unpaired *t*-test (n.s., not significant; *p ≤ 0.05; **p ≤ 0.01; ***p ≤ 0.001).

We tested by luciferase reporter assays whether a 596 bp DNA fragment of the *MKI67* gene can serve as its promoter and mediate cell cycle-dependent transcription (Fig. 3). This fragment included the TSS and all identified conserved elements (Fig. 2 and Suppl. Fig. 2).

We assayed the WT *MKI67* promoter for expression in resting NIH3T3 cells and during progression through the cell cycle. Activity of the *MKI67* upstream region is low in G_0_ and in G_1_ phases. However, the *MKI67* region controls increasing expression of the reporter in S phase, reaching maximum expression when cells progress into G_2_ phase and mitosis (Fig. 3A). This expression pattern is reminiscent of the *MKI67* mRNA expression (Fig. 1A). Therefore, the data suggest that the 596 bp segment serves as the promoter of the *MKI67* gene.

### Two CHR sites and one CDE control *MKI67* cell cycle-dependent transcription

Next, we introduced mutations into the CHRdist, CHRprox, and CDE sites to generate the 3mut construct. These combined mutations led to a complete deregulation of cell cycle-dependent promoter activity (Fig. 3A). Although the activity of the WT *MKI67* promoter construct was low in G_0_ cells, luciferase expression controlled by the 3mut construct was already at a high level. This expression level remained essentially the same throughout the cell cycle for the 3mut reporter, whereas in the same experiment the WT promoter directed the typical cell cycle-dependent expression (Figs. 1B and 3A). The deregulation in the 3mut construct also led to a lower maximal expression in G_2_ phase and mitosis when compared to the WT reporter (Fig. 3A). Taken together, the results suggest that the combination of two CHR elements and a CDE site are responsible for directing cell cycle-dependent transcription of the *MKI67* gene.

A more detailed analysis with individually mutated sites revealed the contribution of single elements. We compared the activities in G_0_ vs. G_2_/M cells of the WT *MKI67* promoter with the activities of various mutant promoters in NIH3T3 cells (Fig. 3B, C). None of the individual mutations of the CHRprox, CHRdist, and CDE sites led to the nearly total loss of regulation that was observed with the triple-mutant 3mut construct. Even combined mutation of two of the three relevant sites in the 2CHRmut and CDE/CHRdist constructs did not yield a complete loss of regulation (Fig. 3B, C). In summary, these results show that the CHRprox, CHRdist, and CDE sites can largely substitute for each other.

However, there is one aspect in which they cannot function alternatively. Promoter constructs with mutations in CHR elements only reached lower maximal activities in G_2_/M cells than their WT and CDE mutant counterparts (Fig. 3A, B). This observation is consistent with transcriptional activation by FOXM1/B-MYB/MuvB complexes through CHR sites, which do not require binding to CDE or E2F elements [39].

Mutation of the three CCAAT-boxes individually and in combinations did not significantly alter cell cycle-dependent transcription from the *MKI67* reporter. Only the overall activity from the promoter reporter decreased upon mutation of CCAAT-boxes (Suppl. Fig. 3).

In summary, the combination of CHRdist, CDE, and CHRprox is responsible for cell cycle-dependent transcription of the *MKI67* promoter.

### DREAM, E2F, and FOXM1/B-MYB/MuvB complex components bind to the *MKI67* promoter in vivo and in vitro

**Fig. 4:**
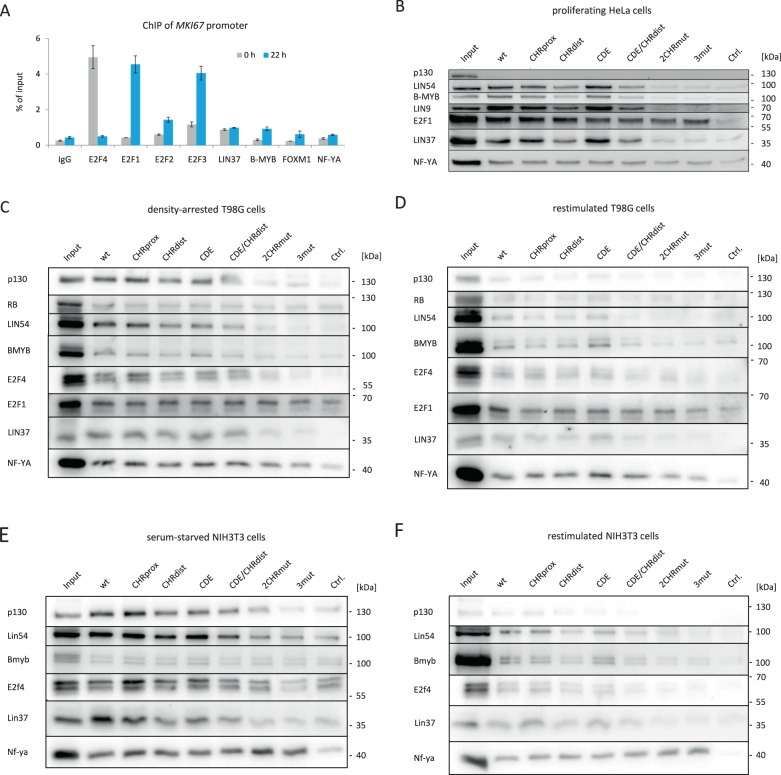
DREAM, E2F, and FOXM1/B-MYB/MuvB complex components bind to the *MKI67* promoter in vivo and in vitro. E2F4 and p130 were detected as representatives of DREAM. B-MYB is specific for B-MYB-MuvB. NF-YA is a subunit of NF-Y. E2F1/2/3 proteins are detected to represent E2F proteins independent of DREAM. FOXM1 detection indicates FOXM1-MuvB binding. LIN37 and LIN54 are representative components of all MuvB-based complexes. **A** In vivo protein binding to the *MKI67* promoter in serum-starved (0 h, gray) and restimulated (22 h, blue) human T98G cells was analyzed via chromatin immunoprecipitation (ChIP) followed by real-time qPCR. Mean values ± SD are given of one representative experiment with two technical replicates for each precipitate. **B**–**F** Nuclear extracts from proliferating HeLa cells (**B**), density-arrested human T98G cells (**C**), restimulated T98G cells (**D**), serum-starved mouse NIH3T3 cells (**E**), as well as restimulated NIH3T3 cells (**F**) were subjected to DNA affinity purification, to assay in vitro binding of complex components by western blot analysis. Fragments of the *MKI67* promoter containing wild-type (wt) or mutant promoter fragments with mutations in CHRprox, CHRdist, and CDE sites were used for DNA probes. 2CHRmut, CDE/CHRdist, and 3mut mutant probes carry combined mutations in CHRdist/CHRprox, CDE/CHRdist, and CHRdist/CHRprox/CDE, respectively. As a negative control of background binding (Ctrl.), a fragment of the mouse *Gapdh2* promoter was employed. Input samples were taken from the nuclear extract before purification. For detection with one specific antibody, all samples were run on the same gel and analyzed via western blotting.

After identifying the regulatory sites in the *MKI67* gene, we investigated protein binding to the promoter. In vivo binding was assayed by ChIP in T98G cells synchronized in G_0_ or G_2_/M (Fig. 4A). We found that E2F4, E2F1/2/3, LIN37, B-MYB, FOXM1, and NF-YA bind to the *MKI67* promoter region.

E2F4, representing the DREAM repressor complex, bound significantly in G_0_ cells, but only weakly in G_2_/M cells. In contrast, the activating transcription factors E2F1/2/3, B-MYB, and FOXM1 displayed elevated binding to the *MKI67* promoter in later phases of the cell cycle but low binding in resting cells (Fig. 4A). Consistent with its role in both repressing and activating MuvB complexes, LIN37 bound similarly well in resting and G_2_/M cells.

NF-YA is a subunit of the hetero-trimeric transcriptional activator NF-Y, which binds CCAAT-boxes [48]. In ChIP assays, we observed that binding of NF-YA in resting vs. G_2_/M cells was not substantially different (Fig. 4A). Taken together with the result that *MKI67* CCAAT-boxes have only a small impact on cell cycle regulation (Suppl. Fig. 3), these observations indicate that NF-Y binding to CCAAT-boxes does not substantially contribute to cell cycle-dependent regulation of *MKI67*.

In conclusion, the results from the ChIP experiments are consistent with downregulation of *MKI67* by DREAM in resting cells and activation by MuvB/B-MYB/FOXM1 in later cell cycle phases.

### DREAM/MuvB complexes bind to both CHR sites in the *MKI67* promoter

To test for differences in protein binding to WT and mutant *MKI67* promoters, DNA affinity purifications were performed (Fig. 4B–F). Nuclear extracts derived from proliferating HeLa cells, density-arrested human T98G cells, restimulated T98G cells, serum-starved mouse NIH3T3 cells, as well as restimulated NIH3T3 cells were employed. Serum-starved or density-arrested cells are largely in G_0_, which allows for binding analysis of DREAM components. In contrast, HeLa cells do not form DREAM or E2F/RB repressor complexes due to the expression of the human papilloma viruses E7 protein, which binds RB, p107, and p130 [43, 49, 50]. However, HeLa cells assemble activator MuvB complexes with B-MYB.

LIN54 and LIN37 proteins were analyzed as indicators for all MuvB-derived complexes. In all assays, binding of LIN54 and LIN37 dropped to the level of the negative control when both CHR sites were destroyed as with the 2CHRmut and 3mut probes (Fig. 4B–F). However, mutation of a single site as in the CHRprox and CHRdist probes still allowed for binding of the MuvB proteins. Binding of the DREAM components E2F4 and p130 was also lost when both CHR sites were mutated, and the same observation was made for B-MYB as a component of activator MuvB complexes in HeLa cells (Fig. 4B–F). As expected, binding of p130 was not detected when the DNA probes were incubated with HeLa extracts. This is in line with the fact that these cells do not form DREAM (Fig. 4B) [50]. Together, the results indicate that the two CHR sites can independently bind repressing and activating MuvB complexes.

Furthermore, mutation of the CDE site led to only small changes compared to the WT probe in any protein binding (Fig. 4B–F). Importantly, binding of E2F1, as a representative of the activator E2Fs, is not altered substantially upon CDE mutation (Fig. 4B–D). This indicates that the CDE site is not overlapping with a functional E2F element, which was a possibility brought up by sequence comparison (Fig. 2). In addition, there is also no significant loss of E2F1 binding upon mutation of the CHRprox and CHRdist sites, which are the key cell cycle elements (Fig. 4B–D). These results suggest that the activating E2Fs do not bind through the elements that are required for cell cycle regulation.

Another important aspect to evaluate a potential function of activator E2Fs in transcriptional repression of *MKI67* is binding of their complexing partner RB. We searched the ENCODE database for RB (RB1) ChIP binding. There is only background binding of RB to the *MKI67* promoter, whereas some binding is detected at the *MYBL2* gene (Suppl. Fig. 4B, C). In the database entry, substantial and consistent binding of E2F4, FOXM1, and B-MYB to the regulatory region of the *MKI67* promoter is observed (Suppl. Fig. 4B, C).

In regard to function of the transcriptional activator complex NF-Y, in vitro binding of NF-YA displayed only small changes for the *MKI67* promoter mutants tested (Fig. 4B–F), which is consistent with the notion that NF-Y binds to CCAAT-boxes. As a secondary aspect, NF-YA binding can therefore serve as loading control for the experiments (Fig. 4B–F).

In summary, the in vitro data show that MuvB complexes bind through individual CHRdist and CHRprox elements independent of the respective other element. In resting cells, both CHR sites can bind DREAM; in later cell cycle phases, they bind MuvB/B-MYB/FOXM1 complexes. In contrast, RB and E2F1, as a representative of activator E2Fs, do not bind to the regulating CHR and CDE sites. These in vitro results are supported by in vivo ChIP data.

### B-MYB activates the *MKI67* promoter through the two CHR elements

**Fig. 5:**
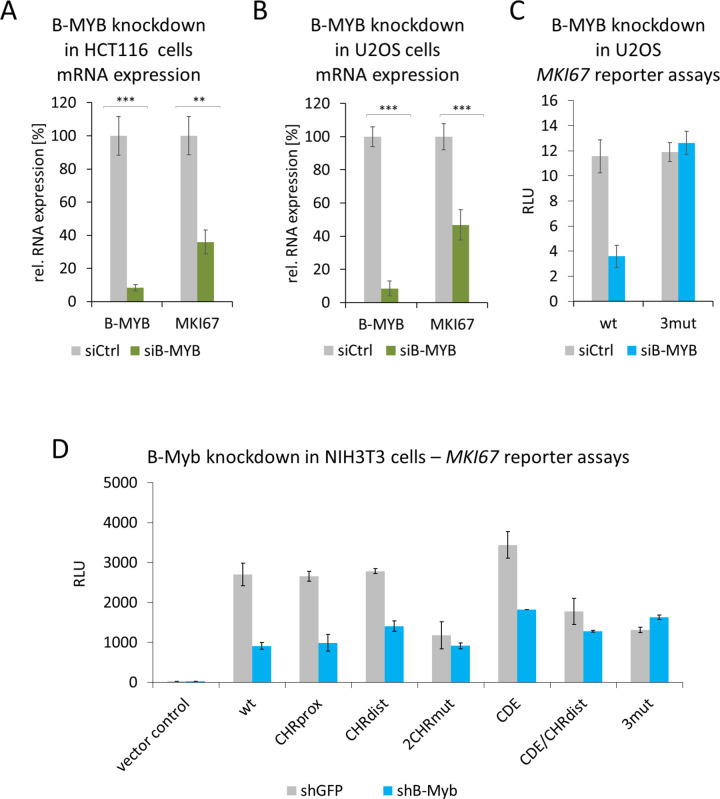
B-MYB activates the *MKI67* promoter through the two CHR elements. **A**, **B** *MKI67* mRNA expression after B-MYB knockdown in HCT116 (**A**) or U2OS (**B**) cells. The cells were transfected with **A** 10 nM or **B** 25 nM non-targeting siRNAs (siCtrl) or B-MYB siRNA (siB-MYB). Relative mRNA levels from *MKI67* and *MYBL2* genes were quantified using real-time RT-qPCR. Mean values ± SD of three (**A**) or four (**B**) biological replicates are given. **C** *MKI67* luciferase promoter reporter assays after B-MYB knockdown in U2OS cells employing wild-type (wt) and mutant *MKI67* promoter (3mut) carrying mutations in the CHRprox, CHRdist, and CDE sites. Mean values ± SD are given of one representative experiment with three technical replicates out of four biological replicates. **D** NIH3T3 cells were transfected with *MKI67* luciferase promoter reporter constructs together with equal amounts of plasmids expressing non-targeting shRNAs (shGFP) or B-Myb shRNAs (shB-Myb). Transfected reporter plasmids were the empty reporter vector pGL4.10 (vector control) and wild-type or mutant reporter plasmid constructs: wild-type (wt), mutants of the proximal CHR (CHRprox), distal CHR (CHRdist), mutant of both CHR elements (2CHRmut), CDE mutant (CDE), a combination of mutations in CHRdist and CDE (CDE/CHRdist), and mutation of the three sites CHRprox, CHRdist, and CDE (3mut). The mean relative light units (RLUs) of two biological replicates and SDs are given.

Next, we tested whether binding of B-MYB correlates with *MKI67* gene activation. In HCT116 and U2OS cell lines, siRNA-directed knockdown of B-MYB yielded a >50% drop in *MKI67* mRNA expression (Fig. 5A, B). Further, B-MYB knockdown substantially reduced WT *MKI67* promoter activity, whereas a reporter mutated in the CHRdist, CHRprox, and CDE sites (3mut) was not affected (Fig. 5C). In addition, transactivation of WT and individual mutant *MKI67* promoter reporters was assayed when B-MYB had been knocked down by shRNAs expressed from cotransfected plasmids (shB-Myb) (Fig. 5D). We found that activity of the WT promoter drops to about one-third in the shB-Myb-treated cells. This effect was lost when both CHR sites were mutated, because the level of activity from those mutant promoters was reduced to about the level of the B-MYB knockdown experiments. In contrast, single mutation of CDE, CHRprox, and CHRdist sites showed the elevated levels of activity. These levels were reduced upon B-MYB knockdown (Fig. 5D).

These results show that both CHR elements are required for full transcriptional activity of the *MKI67* promoter. Furthermore, B-MYB binding to the CHR sites correlates with B-MYB-dependent activation through the two CHR elements.

### DREAM cooperates with RB for maximum repression of *MKI67* gene expression

In experiments employing several knockout cell models, we had shown that DREAM and RB display partially overlapping functions in cell cycle-dependent gene regulation [33, 34]. We had observed that deletion of the DREAM component Lin37 selectively leads to loss of repression by DREAM but does not hamper activation through MuvB complexes [33]. Thus, we were interested in elucidating whether Rb also has an influence on DREAM-dependent control of *Mki67* expression.

**Fig. 6:**
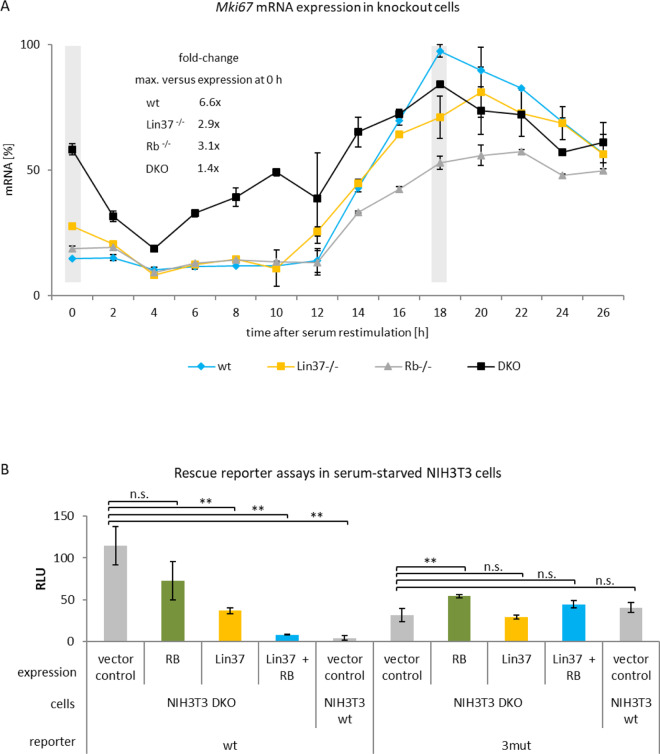
DREAM cooperates with RB for repression of *MKI67* gene expression. Expression of *Mki67* mRNA or expression from *MKI67* reporter constructs in knockout cell models. Wild-type mouse NIH3T3 and knockout cells deficient for Lin37, Rb, or Lin37 and RB (DKO) were employed for the assays. **A** *Mki67* mRNA expression in serum-starved and restimulated cells. Wild-type (wt, blue), Lin37-deficient (*Lin37*^−/−^, yellow), Rb-deficient (*Rb*^−/−^, gray), and DKO (black) cells were arrested by serum starvation (0 h) followed by addition of serum to the medium, to stimulate cells to re-enter the cell cycle. Hours after serum addition are given. Relative *Mki67* mRNA levels were quantified using real-time RT-qPCR. All values were normalized to the highest measurement from the wild-type cells. Mean values ± SD are given of two technical replicates for each time point. The fold-change of *Mki67* mRNA levels was calculated for each cell line by dividing maximal expression by levels at 0 h. Gray bars at 0 and 18 h time points were introduced to emphasize comparison of expression between serum-starved and restimulated cells, respectively. **B** Expression from *MKI67* promoter luciferase reporter constructs and phenotype rescue in cells lacking Lin37 and Rb. Serum-starved NIH3T3 wild-type cells (wt) and NIH3T3 cells deficient for both Lin37 and Rb (DKO) were transfected before starvation with different expression plasmids: empty pcDNA vector (vector control, gray), carrying a cDNA coding for Rb (Rb, green), containing a Lin37 cDNA (Lin37, yellow) or expressing both Lin37 and RB (Lin37 + RB, blue). In addition, cells were transfected with reporter plasmids containing wild-type (wt) or mutated in CHRprox, CDE, and CHRdist elements (3mut) *MKI67* promoter constructs. Luciferase relative light units (RLUs) of one representative out of three biological replicates were measured. Mean values ± SD of three technical replicates are given and significances were calculated using the Student’s *t*-test (n.s., not significant; *p ≤ 0.05; **p ≤ 0.01; ***p ≤ 0.001).

We tested *Mki67* mRNA expression at different time points after serum starvation in WT NIH3T3 mouse fibroblast and knockout cells deficient in Lin37, Rb, or in both Lin37 and Rb (DKO) (Fig. 6A). In WT cells, *Mki67* mRNA expression was low in serum-starved and restimulated cells for up to 10 h in which cells are mostly in G_0_ and G_1_ phases, respectively. Expression increased in S phase, reaching its maximum in G_2_/M (Fig. 6A). In Rb-knockout cells, this expression pattern was similar, but with a small increase in serum-starved cells and a decrease in expression at later restimulation time points, respectively. A more pronounced deregulation was observed in serum-starved Lin37-knockout cells, indicating that DREAM function is more important to repress the *Mki67* promoter in serum-starved cells than Rb function (Fig. 6A). Importantly, the *Mki67* mRNA regulation was largely lost, in particular at early time points, in cells deficient for both Rb and Lin37/DREAM. Still, over the time course, some regulation was observed in the double-knockout cells. However, the substantial deregulation in DKO cells is particularly obvious when focusing on the results for the 0 and 18 h time points (Fig. 6A). In conclusion, both Rb and Lin37/DREAM are important for cell cycle-dependent transcription of *Mki67*. Lin37/DREAM appears to have a larger impact than Rb.

In addition, we performed rescue experiments in the knockout cell lines with *MKI67* promoter reporter assays as readout. We compared regulation of the WT promoter with the 3mut construct, which carries mutations in the two CHR elements and the CDE site. Re-expression of Lin37 and Rb in serum-starved NIH3T3 DKO cells deficient for the two proteins re-established repression of the reporter to a large extent (Fig. 6B). Single rescue experiments by re-expressing Lin37 or Rb alone had some effect compared to the empty vector transfection control experiment. Here again, the effect exerted by Lin37/DREAM appears to be larger than that of Rb (Fig. 6B). The rescue experiments with re-establishing DREAM and Rb/E2F function tested by reporter assays suggested that both protein complexes are required for *MKI67* promoter downregulation through two CHR elements and one CDE site.

Taken together, results from the knockout cell systems indicate that particularly in G_0_ cells, lack of DREAM function has a stronger effect than deletion of Rb on the repression of *Mki67*. Furthermore, the experiments show that Lin37/DREAM and Rb cooperate in downregulating *Mki67* expression.

### Ki-67 downregulation after DNA damage and p53 activation depends on the p53-p21 pathway

**Fig. 7:**
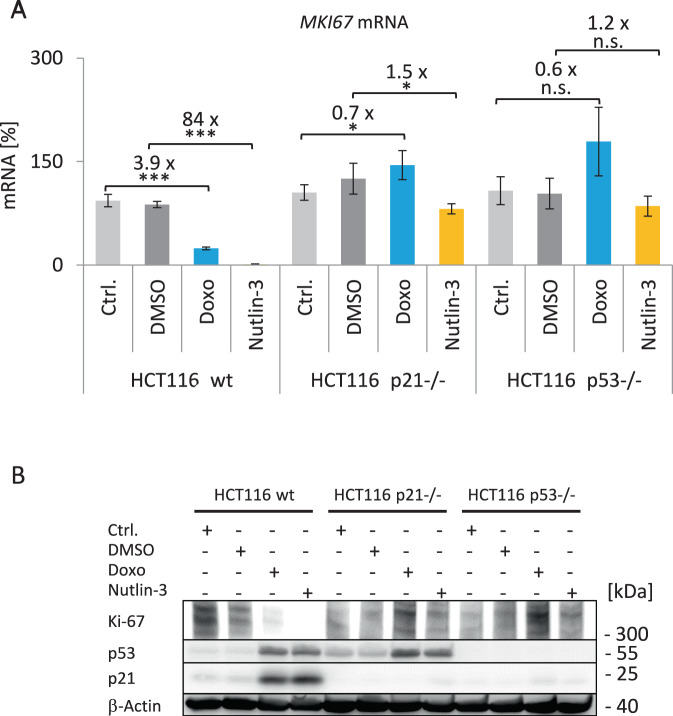
Ki-67 downregulation after DNA damage and p53 activation depends on the p53-p21 pathway. **A** Expression of *MKI67* mRNA in HCT116 wild-type (wt), p21-deficient (*p21*^−/−^), or p53-deficient (*p53*^−/−^) cells treated with doxorubicin (Doxo, blue) or nutlin-3a (yellow) for 48 h. Cells without treatment (Ctrl., light gray) or with DMSO treatment (dark gray) served as controls. *MKI67* mRNA levels are shown, which were quantified from one representative experiment out of two biological replicates by real-time RT-qPCR and normalized to *U6* RNA levels. Mean values ± SD from two technical replicates are given and significances were calculated using the Student’s *t*-test (n.s., not significant; *p ≤ 0.05; **p ≤ 0.01; ***p ≤ 0.001). The relative fold-change in expression levels between treatment and respective control was calculated. **B** Immunoblot detection of Ki-67. Cell samples from the same experiment described in **A** were used for protein extraction. It is one representative experiment of three biological replicates. Ki-67, p53, and p21 protein levels were analyzed via western blotting. β-Actin served as loading control. All samples were run on the same gel and all proteins detected on the same blot.

Following DNA damage, transcriptional repression via the p53-p21-DREAM pathway for genes carrying single CHR elements in their promoters is well established [18, 34]. However, as the *MKI67* gene involves two CHR sites and is also regulated by RB, we examined the effect of p53 and p21 (CDKN1A) induction on *MKI67* expression. To test for regulation by the p53-p21 pathway, we treated human colon carcinoma HCT116 WT, p53-deficient, or p21-deficient cells with Doxo or nutlin-3a to activate p53 signaling (Fig. 7A). In WT cells, *MKI67* mRNA expression was downregulated. However, in *p53*^−/−^ or *p21*^−/−^ cells, *MKI67* downregulation was lost (Fig. 7A). This *MKI67* mRNA pattern is mirrored in Ki-67 protein levels (Fig. 7B). These results show that downregulation of Ki-67 following DNA damage or p53 stabilization requires both p53 and p21. Therefore, Ki-67 appears to be regulated by the p53-p21 pathway.

### LIN37 and RB contribute to *MKI67* mRNA downregulation upon DNA damage induction and p53 activation

**Fig. 8:**
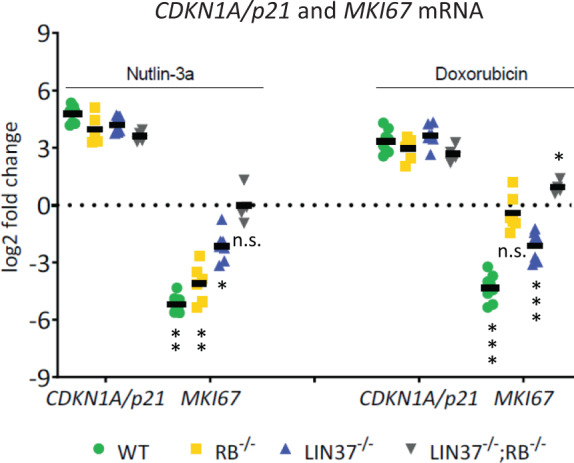
LIN37 and RB contribute to *MKI67* mRNA downregulation upon DNA damage induction and p53 activation. HCT116 wild-type (WT) and mutant cells were tested for mRNA expression of the *MKI67* and the CDK inhibitor *CDKN1A/p21* genes. Clonal cell lines for WT (*n* = 4), *RB*^−/−^ (*n* = 3), *LIN37*^−/−^ (*n* = 4), or double-knockout *LIN37*^−/−^*; RB*^−/−^ (*n* = 2) cells were treated with nutlin-3a or doxorubicin for 48 h. As controls, untreated or DMSO (solvent control)-treated (48 h) cell lines were analyzed. Levels of mRNA from *CDKN1A/p21* and *MKI67* genes were determined by real-time RT-qPCR. The log2-fold changes in mRNA expression of treated vs. control cells are given. Mean values are indicated by black bars. Significances were calculated using the Student’s *t*-test (n.s., not significant; *p ≤ 0.05; **p ≤ 0.01; ***p ≤ 0.001).

After finding that Ki-67 expression is controlled by the p53-p21 pathway, we tested whether this regulation depends on RB or LIN37/DREAM. We tested *MKI67* and *CDKN1A/p21* mRNA expression after nutlin-3a or Doxo treatment in HCT116 WT and *RB*^−/−^, *LIN37*^−/−^, or *LIN37*^−/−^*; RB*^−/−^ mutant cells (Fig. 8). All cell lines tested were positive for WT p53 [51]. Consistently, stabilization of p53 by nutlin-3a or induction of DNA damage by Doxo, respectively, led to a substantial induction of *CDKN1A/p21* mRNA in all cells.

*MKI67* mRNA was downregulated in WT cells by both treatments. Importantly, *MKI67* mRNA repression was completely lost in *LIN37*^−/−^*; RB*^−/−^ double-mutant cells upon p53 stabilization or DNA damage induction (Fig. 8). When treated with nutlin-3a, *MKI67* mRNA downregulation was reduced in RB-deficient cells and even more attenuated in cells deficient in Lin37/DREAM. Upon Doxo treatment, *MKI67* mRNA expression in *LIN37*^−/−^ was still downregulated compared to WT cells, but to a lower extent. Different from the nutlin-3a treatment, incubation with Doxo did not alter *MKI67* mRNA levels in *RB*^−/−^ cells significantly compared to the untreated sample (Fig. 8). These observations show that HCT116 *RB*^−/−^ cells respond differently with *MKI67* mRNA downregulation upon nutlin-3a or Doxo treatment. Taken together, downregulation of *MKI67* mRNA following DNA damage or p53 stabilization depends on intact DREAM and RB.

## Discussion

Cell cycle-dependent expression is the characteristic feature of the Ki-67 protein. Since its discovery, detection of Ki-67 has served as the indicator for proliferating cells [1–4, 52].

Ki-67 protein levels are low or absent in G_0_ and G_1_ phases, and Ki-67 accumulates during S, G_2_, and M phases [53, 54]. Low levels of Ki-67 in G_0_ and G_1_ phases were shown to depend on the timespan that cells have spent in G_0_. Proteasomal degradation has been found to be responsible for Ki-67 loss at the end of mitosis and during G_0_ and G_1_ phases [8, 12, 54]. Importantly, persistent low levels of Ki-67 after G_0_ entry can serve as an indicator for quiescence. Cancer cells, in contrast to normal cells, spend less time in phases between mitosis and re-entering S phase. Therefore, low levels relative to non-detectable Ki-67 expression in G_0_ or G_1_ phases are one indicator of prognosis in malignant diseases [5, 54]. These subtle differences are not detected by standard immunohistochemistry, but more advanced methods allow Ki-67 to become an even better marker of quiescence and proliferation [54].

Although Ki-67 protein expression during the cell cycle has been well studied, reports on transcription and mRNA expression from the *MKI67* gene are limited. Here we show that *MKI67* mRNA expression precedes the appearance of Ki-67 protein with a short lag phase and with similar kinetics (Fig. 1). In reporter assays, we demonstrate that the corresponding kinetics are observed when transcription from the *MKI67* promoter in the cell cycle is examined (Fig. 3). Thus, these results imply that expression of *MKI67* mRNA and subsequent synthesis of Ki-67 protein are dependent on transcriptional regulation from the *MKI67* promoter.

A detailed functional analysis of conserved promoter elements identified two CHR sites responsible for cell cycle-dependent transcription, one of them as part of a CDE/CHR tandem element. Only mutation of all three sites resulted in a near-complete deregulation, indicating that the regulatory elements can in part substitute for each other (Fig. 3). Such a combination of two putative CHR elements is found rarely in G_2_/M-expressed genes and the combination of two functional CHR sites in tandem has not been observed before [21, 23, 39].

**Fig. 9:**
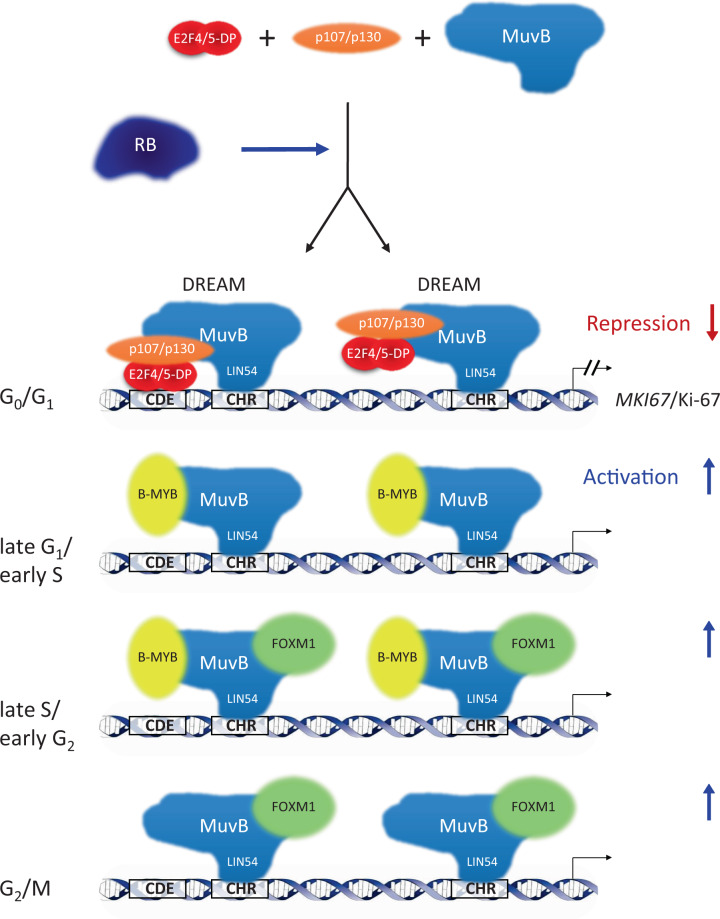
Regulation of *MKI67*/Ki-67 expression during the cell cycle. Repression of transcription in G_0_ and G_1_ cells requires two DREAM complexes binding to one CDE and two CHR sites in the *MKI67* promoter. One DREAM complex binds the downstream CHR site through the LIN54 subunit of the MuvB core complex. The second DREAM complex employs, in addition to a LIN54-CHR binding, complex formation of E2F4/5-DP heterodimers with the CDE site. In contrast to the direct interaction of DREAM complexes with the *MKI67* promoter, RB does not directly control *MKI67* transcription via binding to the gene. RB indirectly contributes to regulation through the CHR and CDE sites by influencing DREAM function. Once cells progress through the cell cycle, the p107/p130-E2F4/5-DP module dissociates from DREAM causing loss of repression. Instead, B-MYB and later FOXM1 sequentially bind to the MuvB core complex. Binding of B-MYB and FOXM1 through LIN54/MuvB to CHR elements leads to transcriptional activation and expression of *MKI67*/Ki-67. Transcription of *MKI67* starts in late G_1_ phase and continues into mitosis with peak expression in G_2_/M.

With the unusual combination of elements, we also observed unexpected combinations of protein binding to the *MKI67* promoter. We showed binding of DREAM/MuvB, B-MYB, and FOXM1 proteins in ChIP assays and in vitro binding experiments through the two CHR sites (Fig. 4 and Suppl. Fig. 4). B-MYB and FOXM1 bind to the MuvB core complex [27, 29]. Importantly, we observe a correlation between B-MYB binding and its function as transactivator. B-MYB-dependent activation requires the two CHR sites in the *MKI67* promoter, which are also required for B-MYB binding (Figs. 4 and 5). Complexes of FOXM1 and B-MYB with MuvB bind DNA through the MuvB component LIN54 and transactivate target genes [20, 55, 56] (Fig. 9). Consistent with the model that MuvB-derived complexes activate *MKI67* transcription through CHR elements, it was reported that expression of Ki-67 as a fusion with a fluorescent protein from endogenous genes was decreased when B-MYB is knocked down [54]. Furthermore, a recent report showed that *MKI67* mRNA is downregulated when FOXM1 is knocked down [57].

In addition to factors regulating through CHR sites, we also found differential binding of the activator E2F proteins E2F1/2/3 by ChIP (Fig. 4). Yet, there are no E2F sites in the *MKI67* promoter and E2F transcription factors are unable to bind through CHR elements [23]. In general, CDE sites are intrinsically related to E2F elements, as they support DREAM binding to CHR sites through binding of the E2F4/5-DP components of DREAM [21, 39]. The CDE upstream of CHRdist overlaps in forward and reverse orientations with a sequence that is close to the E2F-binding consensus (Fig. 2) [47]. However, the activator E2F1 does not bind to this element (Fig. 4).

To contribute to transcriptional repression in G_0_ as observed for *MKI67* expression (Figs. 1 and 6), E2F1/2/3 factors need to form complexes with RB [30]. Therefore, we also examined a potential binding of RB to *MKI67*. However, neither significant binding in vitro to the CDE and CHR elements nor in vivo to the *MKI67* promoter region was observed for RB (Fig. 4 and Suppl. Fig. 4). It is therefore likely that RB does not form complexes with E2F proteins to bind to the *MKI67* promoter to affect *MKI67* expression. Nevertheless, RB has an impact on Ki-67 expression. Thus, we suggest an indirect mechanism that is independent of RB/E2F binding to the *MKI67* gene (Fig. 9). RB can affect cell cycle regulation without binding to E2F promoter sites and without complex formation with activating E2F transcription factors by several mechanisms [25]. Such mechanisms that link RB to the expression or regulation of DREAM components can be phosphorylation, acetylation, ubiquitination, or complex formation of DREAM/MuvB factors as examples. One such functional link between RB and DREAM is exemplified by the RB-LATS2-DYRK1A-LIN52/DREAM axis [58].

In regard to binding of DREAM/MuvB, B-MYB, and FOXM1 proteins, we find binding of the DREAM components E2F4, LIN37, p130, and LIN54, as well as binding of the MuvB-associated factors B-MYB and FOXM1 to the *MKI67* promoter (Fig. 4 and Suppl. Fig. 4). In general, these results on protein binding to the *MKI67* promoter are consistent with earlier observations. In compilations of ChIP data, we noticed that the *MKI67* gene can bind the DREAM/MuvB components p130, E2F4, and LIN9, as well as B-MYB and FOXM1 [17, 18, 29, 59]. In addition, in a screening experiment, it was shown that E2F4 can strongly bind the *MKI67* gene [60]. E2F4 and E2F5, as components of DREAM, can interact with CDE sites, while DREAM attaches to CHR sites with its LIN54 component [39, 61] (Fig. 9).

To elucidate the functional interaction between RB and DREAM, we employed our Rb and Lin37-knockout cell models [33, 34]. Deletion of Lin37 abrogates repressor function of DREAM [33]. We observed that mutation of Rb led to a moderate deregulation of cell cycle-dependent *Mki67* mRNA expression when expression levels in serum-starved vs. restimulated cells were compared (Fig. 6A). Rb-negative cells display an *Mki67* mRNA regulation similar to the WT cells in serum-starved cells. Thus, particularly when comparing regulation in G_0_ cells, Lin37 single mutation caused a larger *Mki67* mRNA deregulation than the Rb mutation. Importantly, mutation of both genes resulted in a substantial loss of regulation. Rescue experiments in serum-starved cells with re-expression of Rb and Lin37 in double-knockout cells also supported the conclusion that both proteins are required for regulation (Fig. 6). These results show that transcriptional repression of *Mki67* depends on DREAM and Rb.

Taken together, all observations are consistent with a model in which MuvB complexes together with RB cooperate in controlling *MKI67* cell cycle-dependent transcription (Fig. 9). DREAM represses transcription in G_0_ and G_1_ phases. *MKI67* downregulation by DREAM is indirectly supported by RB. Starting in S phase, repression of *MKI67* transcription is lost when p107/p130, E2F4/5, and DP dissociate from DREAM/MuvB. Later in the cell cycle, B-MYB or FOXM1 sequentially bind to MuvB, to form the B-MYB-MuvB or FOXM1-MuvB complexes (Fig. 9). These two complexes can activate *MKI67* transcription through two CHR sites in the *MKI67* promoter [19, 20, 27, 30].

Another aspect of *MKI67* transcriptional control is the downregulation of Ki-67 by the tumor suppressor p53 [62]. We showed that indirect p53-dependent downregulation of *MKI67* mRNA and Ki-67 protein requires the cyclin-dependent kinase (CDK) inhibitor p21 (CDKN1A) (Fig. 7). Thus, Ki-67 is a target of the p53-p21-DREAM pathway leading to cell cycle arrest [19, 31]. According to this pathway, p53 activates transcription of *CDKN1A/p21*. The p21 protein then inhibits CDKs that phosphorylate RB family proteins. Members of this family are RB, as well as p107 (RBL1) and p130 (RBL2). Hypophosphorylated p107 or p130 proteins are required to form DREAM. Thus, the p53-p21-dependent formation of DREAM and RB-E2F complexes ultimately downregulates many genes indirectly repressed by p53 [34]. In this large group, Ki-67 downregulation is part of a concerted regulatory program to block the cell cycle through RB and the p53-p21-DREAM pathway [19, 25].

We had previously collected data from genome-wide ChIP studies showing that DREAM complex components bind to *MKI67* [17, 18]. Furthermore, meta-analyses yielded that *MKI67* mRNA is downregulated upon p53 induction in several cell systems [17, 18, 59]. These studies also established that p53 protein does not bind to the *MKI67* gene. In contrast to these observations, it was speculated that binding of Sp1 to p53, resulting in tethering of p53 to Sp1 sites in the *MKI67* promoter, may contribute to repression [63]. However, a tethered Sp1-p53 complex at the *MKI67* promoter is not consistent with the many p53 ChIP experiments, proving that p53 is neither directly nor indirectly bound to the *MKI67* gene [17, 18, 59].

Whether Ki-67 affects the cell cycle has been discussed controversially [10]. Proliferation of cells lacking Ki-67 is largely normal and knockout mice show normal development. However, when exposed to several stresses, Ki-67-knockout cells proliferate less than their WT counterparts [7]. Ki-67 depletion by siRNA causes slower S phase entry compared to untreated cells, results in an increase in p21 (*CDKN1A*) mRNA, and leads to downregulation of G_1_/S genes. Thus, a model was developed that connects downregulation of Ki-67 with an increase in p21, yielding a downregulation of G_1_/S genes, which causes a delay in S phase entry [10]. With our current results, we show that Ki-67 is also a target for downregulation by p21. This observation suggests an amendment to the cell cycle control model by a positive feedback loop, as the downregulation of Ki-67 will lead to a further increase in p21 levels. Also, loss of Ki-67 expression leads to cell stress followed by p53/p21 activation. Furthermore, the model has to be extended, because in addition to G_1_/S also G_2_/M genes are affected by an increase in p21 levels and the resulting repression by DREAM complexes [34].

In addition to proteins such as p21, inhibition of CDKs can also be achieved by small molecule drugs. The CDK inhibitors Palbociclib, Ribociclib, and Abemaciclib are employed in cancer therapy, so far in the treatment of breast cancer. They can inhibit CDK4 and CDK6, leading to hypophosphorylated forms of RB, p107/RBL1, and p130/RBL2. Complex formation with the hypophosphorylated proteins then restores transcriptional repression by RB or DREAM [19, 64–66]. Results presented here suggest that CDK4/6 inhibition leads to *MKI67* downregulation by DREAM. The finding that CDK4/6 inhibitor treatment affects Ki-67 expression prompted the question whether Ki-67 nevertheless remains a reliable parameter to monitor proliferation in a therapeutic setting. It was found that Ki-67 is also a valid marker after such treatments and, consistently, a recent study employed Ki-67 immunohistochemistry to assess patient response following treatment with Abemaciclib [12, 67]. Thus, downregulation of Ki-67 expression through the DREAM-dependent mechanism described here is consolidated by the CDK inhibitors Palbociclib, Ribociclib, and Abemaciclib. Further, as Ki-67 downregulation by CDK inhibitors is accompanied by downregulation of many DREAM targets leading to cell cycle arrest. This links CDK inhibitor function, Ki-67, and DREAM target regulation ultimately with therapeutic success. In consequence, Ki-67 is also a valid indicator for therapy outcome in a setting under CDK inhibitor treatment [19, 30].

In conclusion, we present mechanisms how *MKI67* gene expression followed by Ki-67 protein synthesis is controlled during the cell cycle and upon induction of DNA damage, as well as upon p53 activation.

## Supplementary information

List of Supplemental Information

Suppl. Fig. 1 Quantification of Ki-67 protein

Suppl. Fig. 2 Mapping of the transcriptional start (on genome version hg19)

Suppl. Fig. 3 Contribution of CCAAT-boxes to promoter activity

Suppl. Fig. 4 In vivo Binding of LIN37, E2F, NF-Y, B-MYB, FOXM1, and RB

Suppl. Table 1 - Primer List
