# Supplementary material for: Ki-67 gene expression
Source: Cell Death Differ. 2021 Jun 28;28(12):3357–70. doi: 10.1038/s41418-021-00823-x (PMC8629999; doi:10.1038/s41418-021-00823-x)
Supplement: Supplementary file 1 — List of Supplemental Information [file 41418_2021_823_MOESM1_ESM.docx]

**List of Supplemental Information**

**Supplemental Figure 1**

Quantification of Ki-67 protein.

**Supplemental Figure 2**

Mapping of the transcriptional start (on genome version hg19).

**Supplemental Figure. 3**

Contribution of CCAAT-boxes to promoter activity.

**Supplemental Figure. 4**

*In vivo* Binding of LIN37, E2F, NF-Y, B-MYB, FOXM1, and RB.

**Supplemental Table 1**

List of Primers.
