# Supplementary material for: Ki-67 gene expression
Source: Cell Death Differ. 2021 Jun 28;28(12):3357–70. doi: 10.1038/s41418-021-00823-x (PMC8629999; doi:10.1038/s41418-021-00823-x)
Supplement: Supplementary file 4 — Suppl. Fig. 3 Contribution of CCAAT-boxes to promoter activity [file 41418_2021_823_MOESM4_ESM.pptx]

## Slide 1
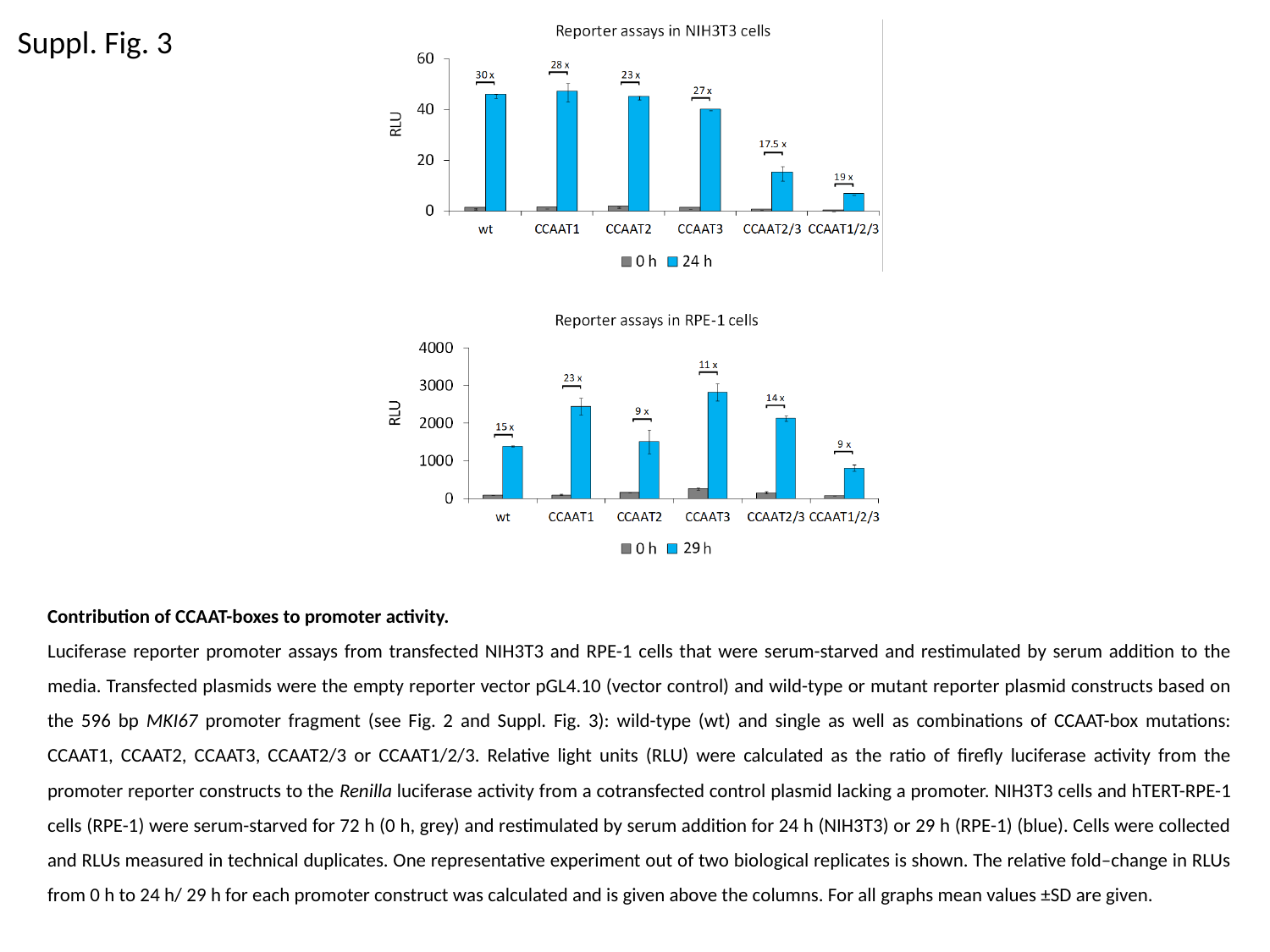

Suppl. Fig. 3
Contribution of CCAAT-boxes to promoter activity.
Luciferase reporter promoter assays from transfected NIH3T3 and RPE-1 cells that were serum-starved and restimulated by serum addition to the media. Transfected plasmids were the empty reporter vector pGL4.10 (vector control) and wild-type or mutant reporter plasmid constructs based on the 596 bp MKI67 promoter fragment (see Fig. 2 and Suppl. Fig. 3): wild-type (wt) and single as well as combinations of CCAAT-box mutations: CCAAT1, CCAAT2, CCAAT3, CCAAT2/3 or CCAAT1/2/3. Relative light units (RLU) were calculated as the ratio of firefly luciferase activity from the promoter reporter constructs to the Renilla luciferase activity from a cotransfected control plasmid lacking a promoter. NIH3T3 cells and hTERT-RPE-1 cells (RPE-1) were serum-starved for 72 h (0 h, grey) and restimulated by serum addition for 24 h (NIH3T3) or 29 h (RPE-1) (blue). Cells were collected and RLUs measured in technical duplicates. One representative experiment out of two biological replicates is shown. The relative fold–change in RLUs from 0 h to 24 h/ 29 h for each promoter construct was calculated and is given above the columns. For all graphs mean values ±SD are given.
