# Supplementary material for: Ki-67 gene expression
Source: Cell Death Differ. 2021 Jun 28;28(12):3357–70. doi: 10.1038/s41418-021-00823-x (PMC8629999; doi:10.1038/s41418-021-00823-x)
Supplement: Supplementary file 5 — Suppl. Fig. 4 In vivo Binding of LIN37, E2F, NF-Y, B-MYB, FOXM1, and RB [file 41418_2021_823_MOESM5_ESM.pptx]

## Slide 1
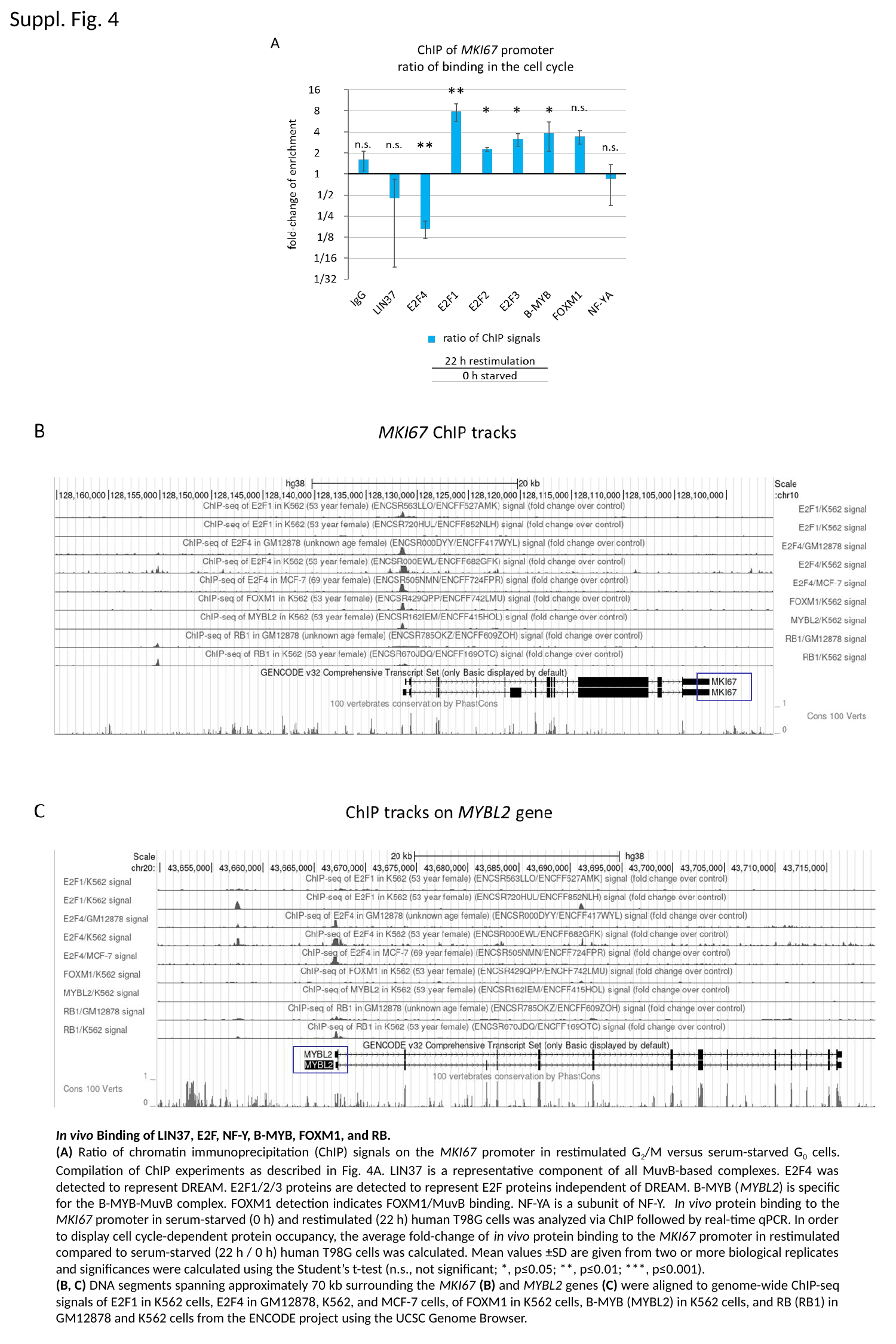

Suppl. Fig. 4
In vivo Binding of LIN37, E2F, NF-Y, B-MYB, FOXM1, and RB.
(A) Ratio of chromatin immunoprecipitation (ChIP) signals on the MKI67 promoter in restimulated G2/M versus serum-starved G0 cells. Compilation of ChIP experiments as described in Fig. 4A. LIN37 is a representative component of all MuvB-based complexes. E2F4 was detected to represent DREAM. E2F1/2/3 proteins are detected to represent E2F proteins independent of DREAM. B-MYB (MYBL2) is specific for the B-MYB-MuvB complex. FOXM1 detection indicates FOXM1/MuvB binding. NF-YA is a subunit of NF-Y. In vivo protein binding to the MKI67 promoter in serum-starved (0 h) and restimulated (22 h) human T98G cells was analyzed via ChIP followed by real-time qPCR. In order to display cell cycle-dependent protein occupancy, the average fold-change of in vivo protein binding to the MKI67 promoter in restimulated compared to serum-starved (22 h / 0 h) human T98G cells was calculated. Mean values ±SD are given from two or more biological replicates and significances were calculated using the Student’s t-test (n.s., not significant; *, p≤0.05; **, p≤0.01; ***, p≤0.001).
(B, C) DNA segments spanning approximately 70 kb surrounding the MKI67 (B) and MYBL2 genes (C) were aligned to genome-wide ChIP-seq signals of E2F1 in K562 cells, E2F4 in GM12878, K562, and MCF-7 cells, of FOXM1 in K562 cells, B-MYB (MYBL2) in K562 cells, and RB (RB1) in GM12878 and K562 cells from the ENCODE project using the UCSC Genome Browser.
